# Measuring the Optimal Time Interval Between Arrival and First Mental Health Evaluation’s for Refugees in Québec: A Scoping Review

**DOI:** 10.1007/s10488-023-01257-y

**Published:** 2023-03-07

**Authors:** Lara Maillet, Anna Goudet, Isabelle Godbout, Gisèle Mandiangu Ntanda, Geneviève Laliberté, France Desjardins, Maryse Benoit, Helen-Maria Vassiliadis, Christine Loignon, Luiza Maria Manceau

**Affiliations:** 1 École Nationale d’Administration publique (ENAP), 4750 av. Henri-Julien, H2T 3E5 Montréal, Québec Canada; 2 Université Laval, Québec, Canada; 3 Université de Sherbrooke, Sherbrooke, Québec Canada; 4 Institut Universitaire de première ligne en santé et services sociaux (IUPLSSS), Sherbrooke, Québec Canada

**Keywords:** Refugees, optimal time interval, mental disorder screening, mental health screening, anxiety disorders screening, psychosocial assessment

## Abstract

To map the state of the existing literature to identify the optimal time frame between the arrival of refugees in a host country and psychosocial assessments. We conducted scoping review using the method of Arksey and O’Malley (2005). A systematic search of 5 databases including PubMed, Psycinfo (OVID), PsycINFO BD APA, Scopus and Web of Sciences) and grey literature identified 2698 references. Thirteen studies published between 2010 and 2021 were considered eligible. A data extraction grid was designed and tested by the research team. It is not so ease to identify the most appropriate time interval to assess the mental health of newly settled refugees. All the studies selected agree on the need to carry out an initial assessment when refugees arrive in their host country. Several authors agree on the need to carry out screening at least twice during the resettlement period. However, what is less clear is the best time to perform the second screening. This scoping review mainly helped in highlighting the lack of probing data on the mental health indicators focused on during the assessment and on the optimal timeline for the assessment of refugees. Further research is needed to determine whether developmental and psychological screening is beneficial, the right time to perform the screening, and the most appropriate collection instruments and interventions.

## Background

The World Health Organization (2016) argues that the interruption of the services continuum for refugees is a major issue that exacerbates their vulnerability, due to their lack of access to the health system, social services and health care providers, and the limited number of services offered. Migrant people, including refugees, are among those with the most unanswered needs in the host society, but they are also among those who are least served by the health system and its practitioners.

Numerous studies on access to primary health and social services support these observations and highlight the difficulties faced by migrant people in obtaining the necessary care, demonstrating that they receive fewer or poorer-quality services; are treated differently by caregivers; and receive treatment that does not meet their needs (Health Canada, 2001; Institut de la statistique du Québec, 2013; Newbold, Cho, & McKeary, 2013).

During the migratory journey that precedes their arrival, many refugees have been exposed to violence and other traumatic events related to war, death, starvation, political violence and forced displacements (Fortuna et al., 2008; Neuner et al., 2010; Rousseau & Drapeau, 2004). Exposure to violence is a major risk factor for the mental health of refugees during the pre-migratory phase and their integration in the host country (Ngo et al., 2000). Mental health is a core aspect of the post-migratory adaptation process (Chu et al., 2013; Mollica, McInnes, Poole, & Tor, 1998; Porter & Haslam, 2005; Schweitzer, Brough, Vromans, & Asic-Kobe, 2011). The migratory experience in itself, including the periods of waiting (in refugee camps or transit countries) and the multiple losses experienced, represents one of the most significant mental health determinants for refugees and can, in certain cases, constitute a traumatic experience (Green et al., 2021; Hassan et al., 2011; Hollifield, Toolson, Verbillis-Kolp, Farmer, et al., 2021; Javanbakht et al., 2019; McArdle & Spina, 2007; Salari, Malekian, Linck, Kristiansson, & Sarkadi, 2017; Shannon, 2014).

The resettlement of refugees during the post-migratory phase can also be a significant source of stress, considering the challenges involved in a sudden adaptation to a new physical and socio-cultural environment (Beiser, Summer 2010; Simich, Beiser, & Mawani, 2003). After such experiences, certain mental health disorders, such as post-traumatic stress disorder, depression, chronic pain and other somatic symptoms appear to a more significant degree among refugees compared to the general population (Kirmayer et al., 2011). Although many refugees can adjust to resettlement in the host country, the responders working with that population should be able to quickly identify any pre-existing mental health disorders that require care, as well as the disorders that could emerge during this time frame, and work to prevent them accordingly (Kirmayer et al., 2011). This vulnerability is exacerbated in particular through the difficulties in accessing health and social services, and due to a restricted number of services offered that are culturally adapted (Shannon et al., 2016; World Health Organization, 2016).

Each year, Canada welcomes an average of 35,000 refugees, of which approximately 6,000 settle in Quebec (Governement of Canada, 2017). However, since November 2015, 31,000 Syrian refugees have added to this number, triggering the formulation of action plans by various governments as well as national social and health departments (Statistics Canada, 2016).

In that regard, the Quebec Department of Health and Social Services developed a Ministerial plan for the assessment of the well-being and physical health status of refugees in a situation of mass arrivals (Ministère de la Santé et des Services sociaux, 2015). It includes a first assessment within 30 days of arrival, in addition to a compulsory health and well-being check-up within 90 days of arrival.

It seems necessary to question and evaluate the relevance of the information gathered in such a short time frame following the arrival of these refugees in Quebec. Numerous responders and actors working with refugees have indeed questioned the relevance of a systematic psychosocial assessment within this time frame. According to their experiences, refugees often present psychosocial problems several months after their arrival in Quebec. At that point, there is no longer a safety net as the assessment has already taken place and such individuals find themselves alone in a complex and difficult to access health and social services system in which the refugees are unfamiliar.

The aim of this scoping review is to map the state of the existing literature to identify the appropriate time frame (time variables) between the arrival of refugees in a host country and psychosocial assessment. Exploring the Canadian and international literature on the topic will put into perspective the plan proposed by the MSSS for Quebec.

## Methods

We conducted a scoping review using the framework of Arksey and O’Malley (2005). A scoping review generally aims to explore a research question by mapping key concepts, types of scientific evidence and research gaps related to a domain or field of research (Arksey & O’Malley, 2005; Levac, Colquhoun, & O’Brien, 2010). This method was conceived for the specific purpose of identifying the gaps in scientific writings and summarizing and sharing the results of synthesis procedures. This method was used to synthesize and share current knowledge on the optimal time frame for collecting information on the mental health of refugees. As suggested by Arksey and O’Malley (2005), specifically, we have: (i) defined the research questions, (ii) selected the relevant articles, (iii) collected the data, (iv) mapped the data, and 5) collated, summarized, and reported the results.

The steps of selecting the keywords, validating the literature, and concluding the scoping review were conducted iteratively, with a view to optimizing the rigour of the process.

### Identifying the Research Question

The research questions were agreed by core research team members (LM, AG, IG and GMN). An overarching research question evolved after discussions as follows: **What is the optimal time interval between the arrival of refugees and their first and follow-up mental health assessments?** Then, the definitions of essential themes for the synthesis were suggested, discussed, and agreed upon within the same core research team members. These themes were “mental health assessment”, “optimal time frame” and “refugees”. This first step helped to clarify the scope of the synthesis and depth of the research (Arksey & O’Malley, 2005; Levac et al., 2010).

### Identifying Relevant Studies

The following steps made it possible to select the papers to be included in the scoping review.

## Data Sources

The identification of the relevant papers and documents were performed in two stages. First, we conducted an electronic search of five databases including PubMed, Psycinfo (OVID), PsycINFO BD APA, Scopus and Web of Sciences. These databases are selected because they list a wide range of scientific papers addressing the topics of interest. Two team members (AG and GMN) developed a search strategy, approved by LM. Consistent with the methodology of the scoping review, we used more sensitive than specific keywords to cover as many papers as possible (Arksey & O’Malley, 2005; Levac et al., 2010). Specifically, we combined the following keywords: mental health screening, indicators and assessment (mental illness screening, mental disorder screening, psychiatric illness screening, anxiety disorders screening, stress disorder screening, depression disorder screening), refugees (migrant, asylum seekers or displaced). Second, other team member (IG) searched additional papers in the list of references of the papers already selected.

## Eligibility Criteria

We included only articles that specifically addressed timing and mental assessment of refugees, published for a period from 2010 to 2021, although we expected to retain a low number of articles. This choice was made to answer the research question as precisely as possible. The exclusion criteria were defined post-hoc in accordance with the methodology of a scoping review (Arksey & O’Malley, 2005; Levac et al., 2010). In a pilot selection phase, AG and GMN read three papers related to our research topic to familiarize themselves with their content. This phase allowed a consensual list of exclusion criteria. Thus, post-hoc, we excluded papers in which:

- the content does not address the time frame of the assessment.
- the content is focusing on internally displaced persons.
- the content is focusing on refugees in camps.
- the content is neither in French nor in English.
- the information on authors is missing.
- the full texts are not available on our institutional databases.

**Fig. 1:**
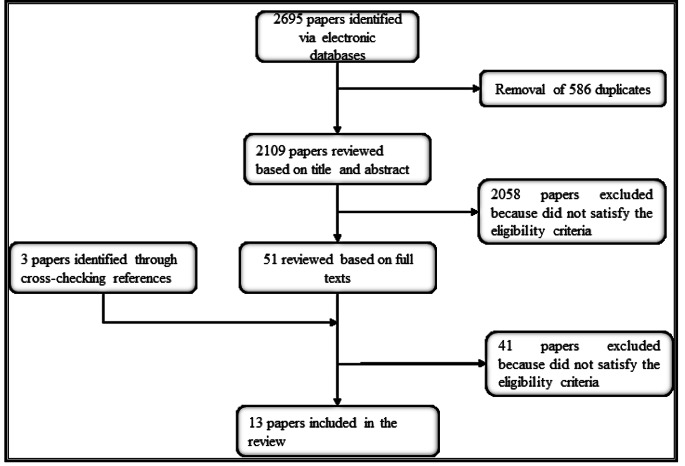
Flow diagram of the selection of the papers

The eligible papers were equally distributed among two teams’ members (AG and GMN), who independently read the titles and abstracts of 20% of the papers assigned to them^1^. Then, a judge (LM) checked the degree of inter-judge agreement between them and each of the three reviewers regarding the 20% of papers read. As the degree of agreement was satisfactory (Kappa greater than 0.65), each reviewer individually finalized the remaining items selection process (80%). The same process was followed for the selection of final papers based on the full texts (Kappa greater than 0.73). Any disagreement that occurred during an item selection stage (titles and abstract, and full texts) was discussed and consensually resolved by AG, GMN and LM. When consensus was not reached, LM took the final decision on whether to include the article or not. A team meeting was held (AG, GMN, IG, LM) during the selection process to discuss the difficulties encountered by the reviewers during this phase and suggest solutions. We used the PRISMA (Preferred Reporting Items for Systematic Reviews and Meta-Analysis) diagram to report the selection process of the items (Fig. 1).

### Study Selection

An iterative data extraction process was developed to address our research question. To extract the data, a grid was designed and tested by the team’s researchers and approved by LM. This grid includes the following data: paper references (author, year), research method, country of study, country of origin of refugees, categories of refugee, population categories (children, adolescents, adults, elderly, pregnant women), time interval of assessment (relative to time of arrival, regularity/frequency, etc.), outcomes related to time interval and other factors influencing assessment (e.g., cultural adjustment, interpreters, life after settlement). For each paper included, we extracted the above elements.

### Charting the Data

The data extraction was carried out in three stages. In the first stage, each publication was categorized based on: type of study, year of publication, country of settlement (Table 1). All papers included were written in English.

**Table 1:** Characteristics of studies included in the review

	Characteristics	Number of papers	%
**Types of the study**	Quantitative study	6	46,2
	Mixed study	2	15,4
	Transversal and/or prospective study	2	15,4
	Longitudinal study	1	7,7
	Review of the literature	2	15,4
**Year of publication**	2010–2015	4	30,8
	2016–2021	9	69,3
**Country of settlement**	United States	7	53,8
	Sweden	2	15,4
	Australia	1	7,7
	Germany	1	7,7
	Switzerland	1	7,7
	Canada	1	7,7

In the second stage, data related to the origin of the refugees (Tables 2 and 3) and target groups (Table 3) were extracted from each paper.

**Table 2:** Countries of origin

Country of origin	Number of papers	%
Irak	9	75
Bhutan	5	42
Burma	5	42
Syria	4	33
Somalia	4	33
Eritrea	3	25
Iran	3	25
Afghanistan	2	17
Lebanon	2	17
Nepal	2	17
Pakistan	2	17
Democratic Republic of Congo	2	17
Albania	1	8
Burundi	1	8
Ethiopia	1	8
Georgia	1	8
Kosovo	1	8
Kurdistan	1	8
Libya	1	8
Macedonia	1	8
Palestine	1	8
Republic of Congo	1	8
Serbia	1	8
Sudan	1	8
Thailand	1	8
Turkey	1	8
Other (African countries, international)	2	17

**Table 3:** Characteristics of studies included in the review

References	Types of study	Countries of establishment	Countries of origin	Target group
Chernet et al. (2021)	Quantitative	Switzerland	Eritrea	Asylum seekers and refugees aged 16 and over who had no major medical problems
Fängström et al. (2019)	Mixed	Sweden	Syria, African countries, Iraq, Turkey, Lebanon, Palestine, Iran, Kurdistan, Pakistan and Thailand	Refugees and asylum seekers, accompanied pre-school children
Green et al. (2021)	Quantitative	United States	Bhutan, Burma, Burundi, Iraq, Somalia	Children refugees aged 4 to 18
Hollifield et al. (2013)	Cross-sectional and prospective analyzes	United States	Bhutan, Iraq, Nepal and Burma	Refugees aged over 14
Hollifield et al. (2021)	Longitudinal study	United States	Bhutan, Iraq and Burma	Refugees over the age of 14
Javanbakht et al. (2019)	Cross-sectional analysis	United States	Syria	Refugees aged 18 to 65
Kaltenbach et al. (2017)	Quantitative	Germany	Syria, Afghanistan, Albania, Kosovo, Iraq, Serbia, Macedonia, Somalia and Georgia	Refugees aged over 12
Salari et al. (2017)	Quantitative	Sweden	Afghanistan, Iran, Syria, Iraq, Pakistan, Somalia, Eritrea, Ethiopia, Libya and Lebanon	Unaccompanied refugees aged between 9 and 18
Polcher and Calloway (2016)	Quantitative	United States	Bhutan, Iraq, Somalia, Congo, Sudan, Burma, Iran, Eritrea	Refugees aged 18 and over
Pottie et al. (2016)	Review of the literature	Mainly Canada and the United States	International	Immigrants, refugees, and asylum seekers (special attention given to refugees and women)
Taylor et al. (2014)	Quantitative	United States	Iraq	Asylum seekers or special immigrant visas, Refugees aged 18 and over
Vukovich (2016)	Mixed	United States	Burma, Iraq, Bhutan/Nepal, Somalia, Democratic Republic of Congo	Refugees aged 18 and over who have met the positive distress criterion within 30 days of arrival
Woodland et al. (2010)	Review of the literature	Australia		Refugees, newly arrived children

Most of the selected articles concern refugees mainly from Iraq, Bhutan, and Burma (Tables 2 and 3) and who are mainly settled in the United States (Table 1).

In the third stage, the results of the selected papers were synthesized (Tables 3 and 4). The following categories were used: study goal and time interval of mental health assessment. The data were analyzed according to main themes of this article: “mental health assessment”, “optimal time frame” and “refugees”.

**Table 4:** Summary of studies included in the review

		Time interval of mental health assessment						
References	Study goal	On arrival	3 months	6 months	9 months	12 months	Follow-ups or references	Other
Chernet et al. (2021)	To assess the mental health status and resilience of Eritrean migrants in Switzerland upon arrival and one-year post-arrival, using standardized mental health screening and resilience assessment tools.	X				X		
Fängström et al. (2019)	To explore the utility of the Strengths and Difficulties Questionnaire (SDQ) with a trauma supplement of six items for preschool children in routine care	X					X	
Green et al. (2021)	To screen refugee children age 4–18 years at their Domestic Medical Examination and three other primary care visits in their first year of resettlement	X		X			X	
Hollifield et al. (2021)	To estimate the prevalence of specific distress trajectory types and identifying important variables that account the variance of the trajectory type in adult refugees of war after resettlement in the U. S							Twice during reinstallation
Hollifield et al. (2013)	To develop empirically a valid, efficient, and effective screening instrument for common mental disorders in refugees	X					X	
Javanbakht et al. (2019)	To determine the prevalence of common consequences of exposure to trauma and high stress, and provide needed interventions, as these conditions if untreated, can be detrimental to mental and physical health	X					X	
Kaltenbach et al. (2017)	To study the feasibility, validity, and reliability of the Refugee Health Screener-15 (RHS-15) - a time-efficient and easy-to-implement screening developed by Hollifield et al. (2013) - as a self-rating and interview instrument	X					X	
Saralri et al. (2017)	To investigate whether a short questionnaire (Children’s Revised Impact of Event Scale; CRIES-8) could be used as a screening tool for PTSD symptoms in URMs, 8–18 years old, during their routine health check-up	X					X	
Taylor et al. (2014)	To assess their physical and mental health status and healthcare access and utilization following the initial 8-month, post-arrival period							After resettlement (approximately one year)
Polcher and Calloway (2016)	Initiate early mental health screening for newly resettled adult refugees at a community health center in Fargo, North Dakota			X			X	
Pottie et al. (2016)	To formulate clinical preventive recommendations to improve patients’ health using an evidence-based clinical preventive approach to complement existing public health approaches							Follow-ups carried out over a period of five years and references
Vukovich (2016)	To explore potential differences in distress levels between newly arrived refugees from Bhutan/Nepal, Burma, Iraq, Somalia, and the Democratic Republic of Congo during the first year of resettlement in Denver, Colorado.	X	X	X	X	X		
Woodland et al. (2010)	To propose a framework for good practice to promote improved access, equity, and quality of care in service delivery for newly arrived refugee children	X					X	

### Collating, Summarizing, And Reporting the Results

Of the 13 publications included in the scoping review, 6 were categorized as quantitative research, 2 as mixed studies, 2 as transversal and/or prospective analysis, 2 as literature review and 1 as longitudinal study. All papers were written in English. The largest number of publications comes from the United States (6), followed by the United Kingdom (2) and Switzerland (2), then Norway (1), Australia (1) and Canada (1). The papers were published between 2010 and 2021, which can be considered an indicator of an emerging field of research or increased interest in the field. The studies targeted different population groups of refugees, immigrants, and asylum seekers: children and adolescents (accompanied or not), adults (with or without a mental health diagnosis) and pregnant women. The countries of origin of the populations under study are very varied, but are mainly concentrated in the Middle East, Africa, and Asia. The countries of settlement of the population under study are mainly in the United States, but also in Sweden, Canada, Australia, Switzerland, and Germany (Table 3).

## Results

Having mapped the information from the studies, we now present a narrative report on the results in two ways. First, we present the results according to the optimal time interval between the arrival of refugees and their first and follow-up mental health assessments. Second, we present the results according to the factors influencing assessment of mental health.

## Timing of the Intervention

The majority of the studies included in this scoping review, most of which are evidence-based studies, recommend carrying out the mental health assessment on arrival and carrying out follow-ups or a referral when mental health problems are detected (Fängström, Dahlberg, Ådahl, Salari, et al., 2019; Hollifield, Toolson, Verbillis-Kolp, Beth Yamazaki, & Tsegaba Holland, 2021; Hollifield et al., 2013; Javanbakht et al., 2019; Kaltenbach, Härdtner, Hermenau, & Schauer, 2017; Salari et al., 2017; Woodland, Burgner, Paxton, & Zwi, 2010). For example, in their publications on health care adapted to refugee children, Woodland et al. (2010) underline the usefulness of a global assessment upon arrival and a follow-up over time: “The key challenges are the provision of both routine comprehensive initial assessment (often requiring specialist skills) and accessible, ongoing care that is universally available to all refugees settling in Australia” (Woodland et al., 2010, p. 565).

A few studies recommend performing the assessment more frequently and at specific intervals. The study of Chernet, Probst-Hensch, Sydow, Paris, and Labhardt (2021) demonstrates that even if they found a trend of improvement in the state of mental health in the population under study one year after the first assessment, one in four participants was still positive for post-traumatic screening stress disorders. For their part, Polcher and Calloway (2016) study stresses the importance of periodic assessments rather than just one screening upon arrival, as mental health is not static, but rather it is dynamic in nature. They recommend performing the mental health assessment at the third clinical visit, approximately six months after the installation:

“New refugees are seen at least 3 times at Family Healthcare in the first 3 to 6 months. […] Often refugees experience a honeymoon period, which is a phase of euphoria that often occurs initially following resettlement. The staff […] have found this period to last from 1 to 3 months for the majority of the new refugees seen at the clinic. Therefore, it was determined that the third visit was the optimal time for mental health screening since most would no longer be in the honeymoon phase” (p. 200).

Among the studies retained, only two carried out the evaluation at several intervals and over a long period. Green et al. (2021) mention that screening upon arrival is essential and emphasize the need for intermittent and continuous screening. After performing screening at different time intervals, including on arrival and after one month, six months and one year, they conclude that practitioners should perform screening on arrival to identify difficulties, and those who have difficulties persisting at six months may need intervention or referral.

For her part, Vukovich (2016) assessed newly arrived refugees five times during the first year of resettlement (30 days, 3 months, 6 months, 9 months and 12 months after arrival) in using different screening tools^2^. Her results indicate that newly arrived refugees from each ethnocultural background exhibit high or low levels of distress and display distinct patterns. Without offering a precise time interval, she mentions that new studies using a longitudinal design are essential to capture individual changes during the first year of resettlement (Vukovich, 2016).

### Factors Influencing the Assessment

This section presents the results relating to the factors (Table 5) that influence the mental health assessment of refugees newly settled in their host country. It should be noted that these results focus solely on mental health assessment and do not extend to the provision of health care globally. The factors identified in this literature review can be classified into four main categories: personal factors, sociopolitical factors, clinical intervention during the assessment and data collection instruments.

**Table 5:** Factors influencing assessment of mental health

References	Personal factors	Socio-political factors	Clinical intervention
Fängström et al. (2019)	Underestimation of the effects of traumatic events Difficulty dealing with traumatic events experienced		When and how to administer the questionnaire Difficulty dealing with traumatic events experienced
Green et al. (2021)	Health profiles vary by country of origin and exposure to stress		
Hollifield et al. (2021)	Migration trajectories		
Hollifield et al. (2013)			Concerns: administration time, workload, and adverse effects on patients
Kaltenbach et al. (2017)	Illiteracy and level of education		Training of clinical staff
Salari et al. (2017)	Illiteracy and level of education	Measures to reduce or regulate the influx of refugees	Offer support in understanding the questionnaire
Polcher and Calloway (2016)	Patient engagement		How to present the screening
Pottie et al. (2016)	Language and other cultural variables Stigma of mental health issues Comorbidity		
Vukovich (2016)	Psychosocial conditions on arrival		
Woodland et al. (2010)	Limited understanding of preventive health care or management of asymptomatic conditions Language and level of education	Limited financial support to monitor the health of refugee children (universal accessibility to care)	Multiple strategies to improve access and quality of care (resources, expertise, public policies, protocols and collaboration)

The personal factors identified concern cultural issues, traumas experienced and migration trajectories, as well as the literacy rate of populations under study. Regarding cultural issues, Woodland et al. (2010) note that refugee families may have limited experience or understanding of preventive health care or the management of asymptomatic conditions. For their part, Fängström, Dahlberg, Ådahl, Salari, et al. (2019) point out that some parents may tend to underestimate the traumatic effects of events experienced by their children. According to Pottie et al. (2011) cultural variations in symptom presentation, coping methods, and the stigma associated with mental health problems can complicate detection and treatment. Many cultures strongly stigmatize mental health issues, which may limit disclosure of behavioural or emotional difficulties experienced. In addition, the presence of significant somatic symptoms, comorbidity, and the tendency of patients to attribute their depressed mood to somatic distress could also reduce symptom recognition (Pottie et al., 2011). Several studies note the need or lack of training for practitioners on refugee health, refugee mental health assessment, and how to address traumatic experiences with refugees (Anude, 2008; Blackmore, 2021; Fängström, Dahlberg, Ådahl, Rask, et al., 2019; Kaltenbach, Härdtner, Hermenau, Schauer, & Elbert, 2017; Shannon, 2014; Woodland et al., 2010).

Regarding migration trajectories, Hollifield, Toolson, Verbillis-Kolp, Beth Yamazaki, et al. (2021) point out that the type of distress trajectory during early resettlement varies by country of origin. Perceived self-efficacy at initial assessment would also protect against reporting initial distress, but other factors such as post-migration stress are more potent in creating distress over time (honeymoon period). Green et al. (2021) point out that refugee children have distinct health profiles depending on the country of origin certainly according to the differences in exposure to stress.

Regarding the literacy level of refugees, Kaltenbach, Härdtner, Hermenau, and Schauer (2017) and Salari et al. (2017) mention that illiteracy and the level of education according to the country of origin should be taken into account when choosing the mode of administration of the screening tools. Salari et al. (2017) report that children with less than four years of schooling had difficulty completing the questionnaire on their own. According to Pottie et al. (2011), the level of underdiagnosis and inadequate treatment is higher among migrants who face cultural, linguistic, and other barriers to accessing mental health care. Language and other cultural variables can hinder accurate diagnostic assessment and treatment .

Regarding sociopolitical issues, only Salari et al. (2017) refer to it. They explain that due to the migratory flow that Europe has been experiencing in recent years, many countries have taken extreme measures aimed at reducing or regulating the influx of refugees. These measures have had the effect of making refugee travel increasingly difficult. Therefore, more recently arrived refugees may have been exposed to more trauma during their journey (Salari et al., 2017).

Regarding the data collection instruments, many authors have identified significant shortcomings in the use of screening tools. First, there would be no “golden standard” (Green et al., 2021). Indeed, a diverse number of questionnaires have been used and some authors point out that these tools have not been validated according to the different populations under study (Pottie et al., 2011).

Thus, Hollifield et al. (2013) insist on the lack of good data on the metric, clinical and social utility of screening which would constitute an obstacle to the development and implementation of screening. They recommend having culturally and linguistically valid instruments. Pottie et al. (2011) point in the same direction. They indicate that the screening instruments have not been culturally validated. Questionnaires are less likely to be accurate due to factors such as language barriers, different cultural norms of behaviour, and different attitudes towards institutional authority (Anude, 2008; Blackmore, 2021; Hollifield et al., 2013; Kaltenbach, Härdtner, Hermenau, Schauer, et al., 2017; Pottie et al., 2011; Vukovich, 2016).

Other authors insist on the limits of certain questionnaires and on the importance of identifying the right threshold of sensitivity according to the population under study. Indeed, according to Taylor, Yanni, Clelia Guterbock, et al. (2014), researchers would overestimate the magnitude of mental health problems when the standard threshold recommended in the literature is used. For their part, Pottie et al. (2011) point out that screening tools tend to have relatively high false positive rates (60–70%) when the prevalence of depression is 10% among immigrants or refugees (Taylor, Yanni, Pezzi, et al., 2014). As for Polcher and Calloway (2016), they point out that the Refugee Health Screener–15 (RHS-15) does not make it possible to assess suicidal ideation and intent to harm oneself or others.

Woodland et al. (2010) interpret the data collection more generally. They explain that a longitudinal survey of immigrants to Australia indicated that a higher proportion of humanitarian entrants compared with other visa streams rated their health as “fair to poor” and had higher levels of health care use, 3.5 years after their arrival. They mention that standardized and consistent data collection across health services, supported financially, would monitor the health of refugee children at the population level and serve to guide service delivery(Woodland et al., 2010).

Furthermore, in addition to assessing mental health, screening tools should be able to assess factors related to the security and stability of newly arrived refugees, such as access to basic needs, English skills, job readiness and social support. These factors would explain the differences in distress levels during the first year of resettlement. Reliable and culturally appropriate screening tools that consider family and community impacts of trauma would be needed to inform planning and service delivery (Vukovich, 2016). Ease of use of the questionnaire for nurses and parents and providing information about the assessment tool and how to use it would also be essential to ensure the quality of the assessment (Fängström, Dahlberg, Ådahl, Salari, et al., 2019).

Finally, various authors mention shortcomings or concerns related to the clinical interventions of health professionals, and more particularly regarding the use of the screening tool. The health professionals who participated in the study of Hollifield et al. (2013) notably mentioned concerns about questionnaire administration time, workload, and possible adverse effects on patients. Others like Salari et al. (2017), mention that the questionnaires can be difficult to understand due to the literacy rate of the respondents. They indicate that clinicians sometimes had to explain certain items of the questionnaire.

Lastly, some authors emphasize how to present and administer the questionnaire. Following their study, Fängström, Dahlberg, Ådahl, Salari, et al. (2019) conclude that the best way to administer the questionnaire is to let the parents fill it out before the health check-up. This allows nurses to prepare before the encounter and use the time to ask follow-up questions and resolve uncertainties. Furthermore, when parents are well informed about the purpose and benefits of the questionnaire, they perceived it as useful (Fängström, Dahlberg, Ådahl, Salari, et al., 2019).

Thus, it is important to train staff to use the questionnaire in culturally appropriate ways to introduce mental health screening, to provide psychoeducation to those who test positive, and to refer refugees to mental health facilities (Kaltenbach et al., 2017). Indeed, the way in which testing is presented to refugees could have a direct impact on the willingness to seek further follow-up (Polcher & Calloway, 2016):

“the attitude and approach by resettlement agencies, case workers, and medical professionals can directly affect refugees’ response to mental health screening when it is presented to them as a tool to assess for emotional distress following resettlement rather than screening for mental illness” (Polcher et Calloway, 2016, p. 202).

## Discussion

Overall, in view of the results of this scoping, it is difficult to answer: **What is the optimal time interval between the arrival of refugees and their first and follow-up mental health assessments?** The results are not binary (yes or no) or quantitative (X months). It is not so ease to identify the most appropriate time interval to assess the mental health of newly settled refugees. Indeed, several questions remain following the study of (Hollifield, Toolson, Verbillis-Kolp, Beth Yamazaki, et al., 2021): “the optimal time to re-screen, identifying people who do not need re-screening, and the public health benefit of screening on health outcomes”. This is why they advocate the importance of early intervention by screening refugees for emotional distress at least twice during resettlement. Woodland et al. (2010) point in the same direction. According to them, further research is needed to determine whether developmental and psychological screening is beneficial, the right time to perform the screening, and the most appropriate collection instruments and interventions.

It should also be mentioned that several authors put forward numerous caveats. First, newly arrived refugees may display distinct trends depending on their ethnocultural background during resettlement (Vukovich, 2016). For their part, Pottie et al. (2011) suggest that children from ethnic minorities are disproportionately over-screened and overreported as positive, particularly for child abuse. Positive false reports could result in harms such as psychological distress, inappropriate family separation, impaired clinician-patient relationship, and legal ramifications associated with child protective services involvement. According to them, systematic screening is not recommended for this clientele.

### Timing of the First Initial Assessment

In general, all the studies selected agree on the need to carry out an initial assessment when refugees arrive in their host country. Some authors (Fängström, Dahlberg, Ådahl, Salari, et al., 2019; Javanbakht et al., 2019; Kaltenbach, Härdtner, Hermenau, & Schauer, 2017) recommend completing the mental health assessment at the same time as the physical health assessment during the first visit. However, as Anude (2008) mentions, the programs and services offered for newly arrived refugees may vary from one city, province or country to another. Thus, discrepancies may exist in screening procedures and practices, particularly regarding the timing of the first assessment.

Several authors agree on the need to carry out screening at least twice during the resettlement period, in particular to detect people suffering from initial distress and those suffering from late or persistent distress (Green et al., 2021; Hollifield, Toolson, Verbillis-Kolp, Beth Yamazaki, et al., 2021). However, as mentioned by Green et al. (2021), what is less clear is the best time to perform the second screening. For his part, Hollifield, Toolson, Verbillis-Kolp, Beth Yamazaki, et al. (2021) recommends performing it six months after resettlement. In addition, some authors insist on the need for intermittent and continuous screening (Green et al., 2021), while others warn against the negative effects of systematic screening in certain clienteles (Pottie et al., 2011). Finally, the honeymoon period experienced by some refugees following resettlement must also be taken into consideration (Polcher & Calloway, 2016).

Based on these findings, we recommend the establishment of a systematic mental health screening program for recently settled refugees. Indeed, according to Pottie et al. (2011), almost all people with major depression are seen only in primary care and nearly 60% of them go undetected and untreated. The level of underdiagnosis and inadequate treatment of depression is higher among migrants who face linguistic, cultural, or other barriers. Among refugee patients with depression, more than half also has post-traumatic stress disorder and this comorbidity can complicate the recognition of depression (Pottie et al., 2011). Some of the most important factors of psychological morbidity among refugees could be mitigated by planned and integrated rehabilitation programs and attention to social support and family unity (Hassan et al., 2011).

The screening program should allow newly settled refugees to receive an initial assessment of their mental health (no later than one month after their arrival) at the first clinic visit and a second assessment six months after resettlement to allow the honeymoon period to pass. The program should provide a central entry point, access to trained interpreters, and the availability of community mental health services (Hollifield et al., 2013). Interdisciplinary and sustainable models of care that support an integrated approach, incorporating multidisciplinary health care providers and coordination of care (Johnson-Agbakwu et al., 2014) should be offered. Clinical professionals should also receive training in evidence-based practices for addressing symptoms of refugee trauma (Anude, 2008; Shannon, 2014) and to administer the questionnaire properly (Fängström, Dahlberg, Ådahl, Salari, et al., 2019). The assessment should be conducted using validated and culturally appropriate screening tools for diverse refugee populations (Hollifield et al., 2013; Pottie et al., 2011; Vukovich, 2016). Finally, a broader national refugee health policy that considers other determinants of health, such as education, housing and employment, would improve long-term health outcomes, adaptation social-emotional and educational achievement of refugees (Woodland et al., 2010).

### Strengths and Limitations

This scoping review has major strengths. First, it includes a review of the scientific and grey literature as part of a systematic and multisource procedure, also validated by a multi-evaluation process. Second, it is based on both Canadian and international literature, considering qualitative and quantitative studies, as well as empirical and review research, covering a wide range of materials produced on the topic. It therefore allows a rigorous process to be undertaken in identifying recommendations for public health institutions. When considering accountability and priorities in terms of health issues, particularly in mental health, it is imperative to show the rationale that led to the formulation of these indicators.

However, this scoping review also has limitations. First, only English and French articles were included; other valuable documents are likely to have been published in other languages. Second, double-checking was not carried out on all of the study extraction procedures. Restricted human resources and time frames also limited the overall scope of the scoping review. Third, also due to human resource and time constraints, the time frame selected (2010–2021) is limitative, as it excludes the significant refugee movements of the 1970–1980 s and the 1990s (including those of the so-called boat people from Southeast Asia, who set a precedent for how refugees in Canada and Quebec went on to be received and welcomed). Fourth, some recommendations were related to macro-level contexts, and consequently were difficult to implement.

Nonetheless, the results have helped in identifying several interesting research opportunities regarding mental health assessments for refugees and immigrants within optimal time frames and adapted to various trajectory contexts of refugees and of host countries.

## Conclusion

Through this study, we wanted to answer the question of what the optimal time interval between the arrival of refugees is and their first and follow-up mental health assessments. This scoping review mainly helped in highlighting the lack of probing data on the mental health indicators focused on during the assessment and on the optimal timeline for the assessment of refugees. This absence of data can be observed both in Canadian and international literature and confirms the need to deepen research in this area to develop better assessment and intervention strategies. Public and other authorities are responsible for making decisions on the time frames between the arrival in the host country, the initial consultation with the health and social service professionals as well as the continuity of services if needed. It is important to document the best practices collated in terms of assessment and optimal time frame with regard to such assessments for refugees. Woodland et al. (2010) come to the same conclusion: “Given the likely interrelationships in producing good health outcomes, further research is required to determine whether developmental and psychological screening is of benefit and the most appropriate timing, instruments and interventions” (Woodland et al., 2010).

Our scoping review provides an unprecedented study of the issue of indicators and optimal time frames for initial mental health assessments for refugees upon arrival in their host countries. In addition to the issue of the time frame, this study also highlighted the other conditions that have an influence on the assessment. Furthermore, our scoping review has shown that there are many interesting research opportunities in connection with mental health assessments for refugees in optimal time frames and adapted to the various trajectories and contexts of refugees and of host countries.

Nonetheless, some questions remain unanswered and should be given further attention. First of all, some authors raise concerns about potential adverse effects of screening. The studies do not question the relevance of screening. They also do not determine whether systematic screening and/or need-based follow-up is preferable. Then, some studies such as that of Polcher and Calloway (2016) state that some patients do not commit to or do not necessarily participate in follow-up consultations. It would be interesting to collect additional data on post-screening follow-up or on the absence of follow-up. Finally, some studies such as Shannon’s (2014) state the advantages and disadvantages of family screening versus individual screening. It would be useful to explore this question further.

Considering that this scoping review includes a wide range of assessment methods and suggests that refugee mental health assessments in optimal time frames could be improved by formal but flexible adaptation of services, future research could provide stronger evidence on more nuanced interventions and other types of outcomes. Future research should also take into consideration the post-pandemic context of COVID-19, which has, among other things, exacerbated economic and health inequalities and insecurities, mainly among refugee populations.

## Data Availability

The datasets analyzed during the current study are available from the corresponding author on reasonable request.

## Footnotes

1 The fact that two members of the research team were unable to read the titles and abstracts of all the articles constitutes a limit to our study. It is explained in the Strengths and Limitations section of the article.

2 To assess the mental health of refugees, the authors of the articles included in this scoping review used different types of assessment tools including administered or self-administered questionnaires. Among the most frequently used questionnaires: the Refugee Health Screener (RHS), the Patient Health Questionnaire (PHQ), the Posttraumatic Stress Disorder Checklist (PCL) and the Strengths and Difficulties Questionnaire (SDQ).
